# Genome sequence of *Methylocystis hirsuta *CSC1, a polyhydroxyalkanoate producing methanotroph

**DOI:** 10.1002/mbo3.771

**Published:** 2018-12-12

**Authors:** Sergio Bordel, Elisa Rodríguez, Raúl Muñoz

**Affiliations:** ^1^ Departamento de Ingeniería Química y Tecnología del Medio Ambiente, Escuela de Ingenierías Industriales Universidad de Valladolid Valladolid Spain; ^2^ Institute of Sustainable Processes Universidad de Valladolid Valladolid Spain

**Keywords:** genome, methane monooxygenases, methanotrophs, methylocystis, polyhydroxyalkanoates

## Abstract

Polyhydroxyalkanoates (PHAs) are biodegradable plastics that can be produced by some methanotrophic organisms such as those of the genus *Methylocystis*. This allows the conversion of a detrimental greenhouse gas into an environmentally friendly high added‐value bioproduct. This study presents the genome sequence of *Methylocystis hirsuta *CSC1 (a high yield PHB producer). The genome comprises 4,213,043 bp in 4 contigs, with the largest contig being 3,776,027 bp long. Two of the other contigs are likely to correspond to large size plasmids. A total of 4,664 coding sequences were annotated, revealing a PHA production cluster, two distinct particulate methane monooxygenases with active catalytic sites, as well as a nitrogen fixation operon and a partial denitrification pathway.

## 1 INTRODUCTION

Anthropogenic emissions of methane currently account for up to 30% of the global emissions of greenhouse gases (considering that methane has a 25 times greater global warming potential than CO_2_; Desai & Harvey, 2017). Despite the fact that methane can be used as an industrial energy source via combustion at concentrations higher than 20%, more than 56% of its emissions have concentrations lower than 5% (Lebrero et al., 2016). Biological methane abatement is a very attractive alternative to treat diluted methane emissions based on its high effectiveness and environmentally friendliness. In addition, biological methane abatement can be coupled to the production of added‐value compounds. Polyhydroxyalkanoates (PHAs) are intracellular biopolyesters produced under nutrient‐limiting conditions by a wide range of methane‐consuming organisms (Pieja, Morse, & Cal, 2017).

Methanotrophs are organisms able to use methane as the sole energy and carbon source, some of them use methane exclusively and others are facultative methanotrophs, able to grow also in other carbon sources. We focus here on bacterial methanotrophic species using oxygen as electron acceptor. Even if anaerobic methane oxidation can also occur coupled to sulfate, nitrate and nitrite reduction, this phenomenon plays a minor ecological role compared to aerobic oxidation. Aerobic methanotrophic bacteria are usually classified into type I and type II methanotrophs, which are different in their membrane arrangement, fatty acid composition, and methane assimilation pathways (Hanson & Hanson, 1996). Type II methanotrophs such as *Methylocystis*, *Methylosinus,* and *Methylocella* are considered the main methanotrophic PHA‐synthesizing genera. For instance,* Methylocystis hirsuta *has been shown to accumulate PHB up to 45% of its total biomass (García‐Pérez et al., 2018), which makes it a very interesting cell factory. This value is higher than those previously reported for other methanotrophs (Pieja, Rostowski, & Criddle, 2011), including its close relative *Methylocystis *sp. SC2*.*

## 2 MATERIALS AND METHODS

The strain *M. hirsuta *CSC1, which was obtained from DSMZ (DSM no. 18500) (Lidner et al., 2007), was cultured in 1,250 ml gas‐tight bottles containing 210 ml of a mineral salt medium (Mokhtari‐Hosseini et al., 2009). Methane was then supplied at an initial headspace concentration of 195 ± 7 g/m^3^ (under O_2_ sufficient conditions), and the bottles were incubated in an orbital shaker (MaxQ 4000; Thermo Scientific, USA) at 30°C and 200 rpm for a week. Biomass was centrifuged, and sequencing libraries were prepared (after checking the purity of the culture by 16S sequencing) using the protocol for multiplexed microbial SMRTbell libraries for the PacBio Sequel System. The genetic material was fragmented and selected to have a size close to 20 kb. The final size distribution was checked using AATI Femto Pulse. The library was sequenced using the platform Sequel from PacBio, with an acquisition time of 10 hr. The sequenced reads were assembled using HGAP 4.0 (Chin et al., 2013).

## 3 RESULTS

The assembly's results are summarized in Table 1.

**Table 1:** Genome statistics of *Methylocystis hirsuta CSC1*

Genome feature	Value
Size (bp)	4,213,043
Contigs	4
N50 (bp)	3,776,027
L50	1
GC content (%)	62.4
Coding sequences	4,664
Number of RNAs	50

**Figure 1:**
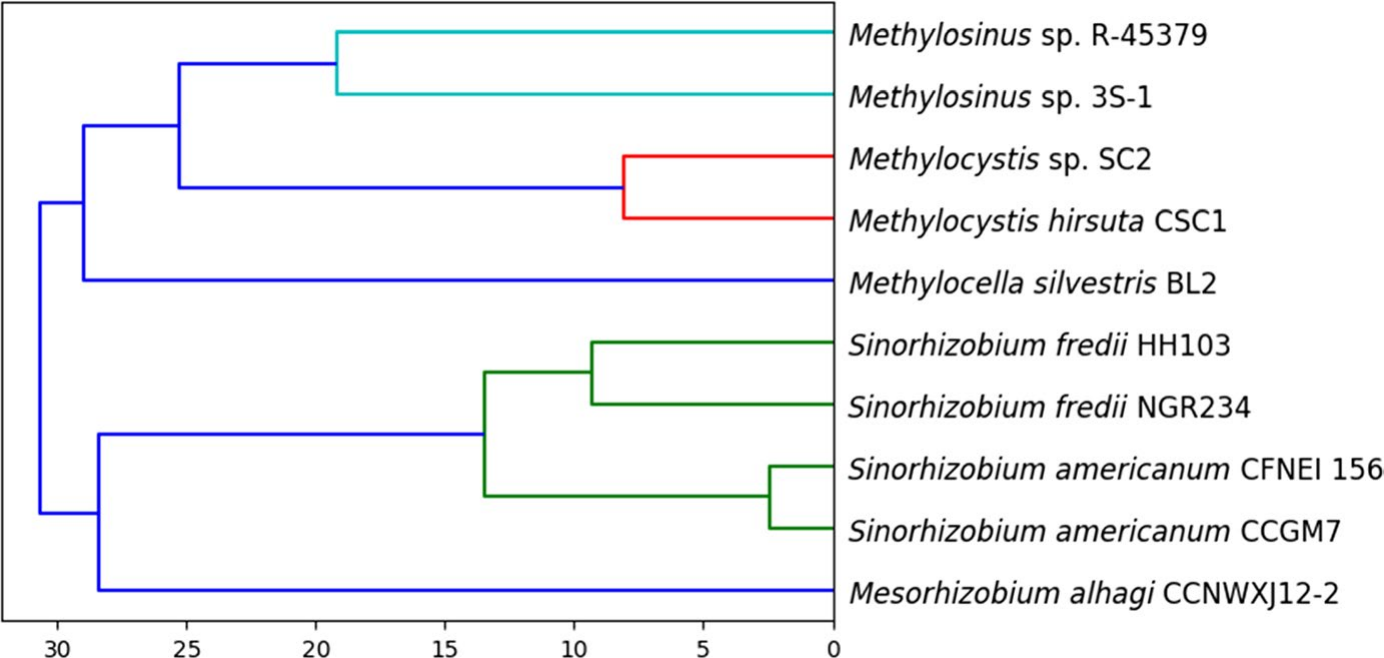
Phylogenetic relations of *Methylocystis hirsuta* CSC1. The *x*‐axis represents 100 minus the average nucleotide identity among species. The closest related species was *Methylocystis *sp. CS2. *Methylosinus* appeared to be the closest genus to *Methylocystis*

A phylogenetic analysis was carried out using JSpeciesWS (Richter, Rosselló‐Móra, Glöckner, & Peplies, 2015), which calculates the average nucleotide identity (ANI) comparing all shared orthologous protein‐coding genes of two genomes (Richter & Rosselló‐Móra, 2009). The phylogenetic tree (Figure 1) containing the 10 closest species identified by JSpeciesWS was built using the function *dendrogram* from SciPy after defining the distance between species as 100 minus their ANI value.

The genome was annotated using the NCBI Prokaryotic Genome annotation pipeline (Tatusova et al., 2016). A general summary of the biological functions coded in the genome was obtained using RAST (Overbeek et al., 2014). Approximately, 25% of the annotated genes corresponded to RAST subsystems (Table 2).

**Table 2:** Biological subsystem distribution of annotated genes in *Methylocystis hirsuta* CSC1

Code	Description	Value	Percentage
A	Cofactors, vitamins, prosthetic groups, pigments	143	8.8
B	Cell wall and capsule	47	2.9
C	Virulence, disease and defense	77	4.7
D	Potassium metabolism	7	0.4
E	Miscellaneous	37	2.3
F	Phages, prophages, transposable elements, plasmids	41	2.5
G	Membrane transport	75	4.6
H	Iron metabolism	0	0
I	RNA metabolism	49	3
J	Nucleosides and nucleotides	51	3.1
K	Protein metabolism	187	11.5
L	Cell division and cell cycle	0	0
M	Motility and chemotaxis	0	0
N	Regulation and cell signaling	34	2.1
O	Secondary metabolism	7	0.4
P	DNA metabolism	76	4.7
Q	Fatty acids, lipids, and isoprenoids	68	4.2
R	Nitrogen metabolism	39	2.4
S	Dormancy and sporulation	1	0.1
T	Respiration	126	7.7
U	Stress response	70	4.3
V	Metabolism of aromatic compounds	13	0.8
W	Amino acids and derivatives	243	14.9
X	Sulfur metabolism	13	0.8
Y	Phosphorous metabolism	17	1
Z	Carbohydrates	184	11.3

Despite the presence of plasmids has not been assessed experimentally, a cluster of three plasmid replication genes (*RepA*, *RepB* and *RepC*) was detected in the fourth contig (which is 158,363 bases long). This suggests that this contig could correspond to a large plasmid. The locus tags of the genes forming this cluster of plasmid replication genes are as follows: D1030_20715, D1030_20710, and D1030_20705. The fourth contig also contains several repeat regions that could be binding sites for the plasmid‐encoded repeat proteins (Sekine et al., 2006). The third contig (260,028 bp) contains also two plasmid replication genes (*RepA* and *RepC*) separated by a protein that could not be annotated and has repeated regions in their proximity, which suggests the possibility of this contig being also a large size plasmid. The chromosome also contains two genes annotated as RepA proteins and two other putative RepC proteins. Overall, it appears very likely that *M. hirsuta *is able to sustain plasmid replication. The closest strain *Methylocystis *sp. SC2 does contain two large plasmids.

**Figure 2:**
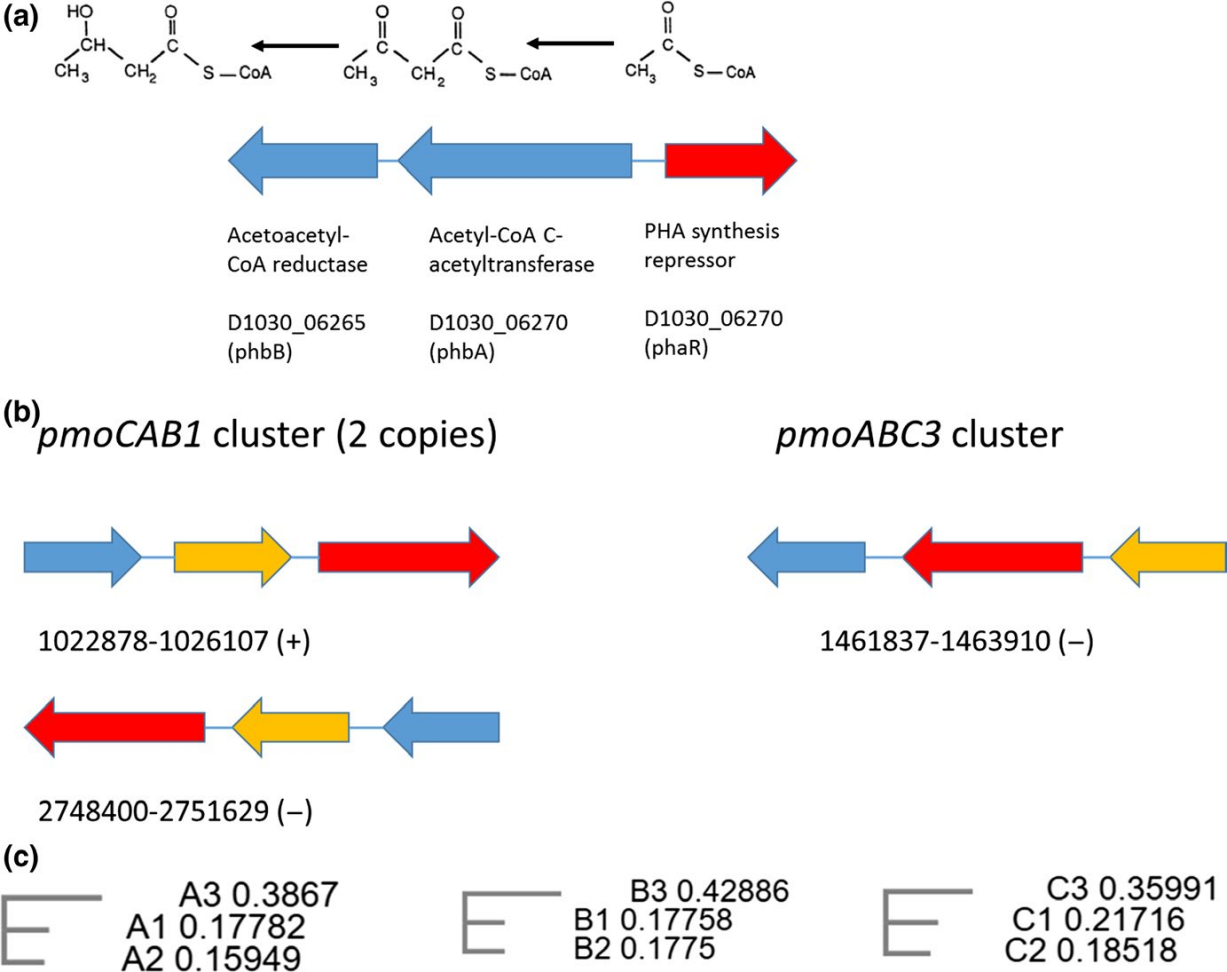
PHB synthesis and methane oxidation cluster. (a) PHB synthesis cluster. (b) Two clusters coding pMMO enzymes. The *pmoCAB1 *cluster is duplicated and is identical to the cluster found in *Methylocystis *sp. SC2. The *pmoABC3* cluster differs in the order in which the subunits are arranged. (c) Evolutionary distance between proteins in the *pmoCAB1*, *pmoABC3,* and *pmoCAB2 *clusters. *pmoCAB2* is present in *Methylocystis *sp. SC2 but is absent in *Methylocystis hirsuta *CSC1

In order to identify gene clusters involved in the synthesis of secondary metabolites, the platform antiSMASH 4.0 was used (Blin et al., 2017). The two clusters showing higher similarity (to known clusters) were a PHA biosynthetic gene cluster and an enterobactin biosynthetic cluster (Table 3). The PHA biosynthetic cluster (Figure 2a) contains the genes phbA, phbB and the regulator phaR. The gene involved in the last step of PHB biosynthesis (D1030_08315) is outside of this cluster. The same arrangement of phbA, phbB, and phaR is observed in the strain *Methylocystis *sp. SC2 (Dam, Dam, Kube, Reinhardt, & Liesack, 2012). On the other hand, enterobactin is a chelating agent that has a strong affinity for iron and is secreted to the environment to improve iron assimilation. A cluster of eight genes involved in the synthesis of enterobactin from chorismate was found. This cluster has the same architecture in *Methylocystis *sp. SC2, which suggests that these organisms are able to uptake iron with high efficiency.

**Table 3:** Enterobactin biosynthesis and transport genes in *Methylocystis hirsuta *CSC1

Locus tag	Genomic coordinates	Annotation
D1030_04505	893002–894342 (+)	Enterobactin esterase
D1030_04515	894639–901834 (+)	Siderophore biosynthesis non‐ribosomal peptide synthetase modules
D1030_04520	901841–903292 (+)	Enterobactin exporter EntS
D1030_04525	903318–904544 (+)	Isochorismate synthase
D1030_04530	904535–906157 (+)	2,3‐hydroxybenzoate‐AMP ligase
D1030_04535	906177–907064 (+)	Isochorismatase
D1030_04540	907082–907828 (+)	2,3‐dihydro‐2,3‐dihydroxybenzoate dehydrogenase

*Methylocystis* *hirsuta *CSC1 contained two different gene clusters coding the three subunits (A, B, and C) of particulate methane monooxygenase (pMMO) enzyme, one of them was present in two copies (Figure 2b) and was identical to the *pmoCAB1* cluster present in *Methylocystis *sp. SC2 (Dam et al., 2012). One of the copies in *M. hirsuta* CSC1 was oriented in the reverse sense compared to *Methylocystis *sp. SC2 as a result of a chromosomal inversion. The pMMO coded by the* pmoCAB1* cluster has been reported to oxidize methane at gas concentrations higher than 600 ppm_v _(Baani & Liesack, 2008). *Methylocystis *sp. SC2 contains a second pMMO cluster, *pmoCAB2*, which codes an enzyme with higher affinity for methane at low concentrations. This cluster was absent in *M. hirsuta *CSC1, which contained instead a second cluster (Figure 2b) with the pMMO subunits arranged in the order ABC and that is designated as *pmoABC3* cluster. In order to identify the evolutionary relations among these three gene clusters, a multiple alignment of each of their proteins was performed using the software MUSCLE (Edgard, 2004). Figure 2c shows that the proteins in the *pmoABC3 *cluster are more evolutionary distant than those in the two other clusters (*pmoCAB1 *and *2*). A BLAST search revealed that the *pmoABC3* cluster can be also found in *M. hirsuta *SB2 (Vorobev et al., 2014) with a 96% nucleotide identity. *M. hirsuta *SB2 actually contains all the 3 pMMO clusters discussed previously. Finally, the catalytic sites of all the pMMO subunits in both clusters are well conserved, which suggests the existence of two active pMMOs that could be tailored to work under different environmental conditions.

The genome sequence contained two malyl‐CoA lyases (D1030_00725 and D1030_16225), which suggests that *M. hirsuta* CSC1 uses the serine cycle to assimilate C1 compounds, similarly to most type II methanotrophs (Hanson & Hanson, 1996).

A complete *nif *operon (involved in nitrogen fixation) was found (with genomic coordinates 818892–827543). The operon contained the *nifH, D, K, E, N,* and *X* genes. This suggests that *M. hirsuta *CSC1 was able to fix atmospheric nitrogen similarly to *Methylocystis *sp. SC2 (Dam, Dam, Blom, & Liesack, 2013). *M. hirsuta* CSC1 contained also genes involved in denitrification. However, no nitrous oxide reductases were annotated (in contrast to *Methylocystis *sp. SC2 that contains an operon with six genes involved in N_2_O reduction). Therefore, *M. hirsuta *CSC1 possesses the potential to perform only a partial denitrification, with an associated production of N_2_O under anaerobic conditions.

## CONFLICT OF INTEREST

The authors declare not to have any conflict of interest.

## AUTHORS CONTRIBUTION

SB performed the bioinformatics analysis and wrote the manuscript. ER carried out the microbial cultures and sample preparation. RM conceived and supervised the work. All the authors edited the manuscript.

## ETHICS STATEMENT

None required.

## Data Availability

This whole genome shotgun project has been deposited at DDBJ/ENA/GenBank under the accession number QWDD00000000 (BioProject: PRJNA487728).
